# Biocompatibility and antibacterial properties of zirconium nitride coating on titanium abutments: An *in vitro* study

**DOI:** 10.1371/journal.pone.0199591

**Published:** 2018-06-26

**Authors:** Giulia Brunello, Paola Brun, Chiara Gardin, Letizia Ferroni, Eriberto Bressan, Roberto Meneghello, Barbara Zavan, Stefano Sivolella

**Affiliations:** 1 Department of Management and Engineering, University of Padova, Vicenza, Italy; 2 Department of Neurosciences, Section of Dentistry, University of Padova, Padova, Italy; 3 Department of Molecular Medicine, University of Padova, Padova, Italy; 4 Department of Biomedical Sciences, University of Padova, Padova, Italy; 5 Maria Cecilia Hospital, GVM Care & Research, E.S: Health Science Foundation, Cotignola, Italy; VIT University, INDIA

## Abstract

Improving soft tissue attachment and reducing bacterial colonization on titanium abutments are key factors for the long-term maintenance of healthy soft and hard peri-implant tissues. This *in vitro* study was conducted to compare the biocompatibility and antibacterial activity of four different surfaces: uncoated Ti6Al4V, anodized, and coated with titanium nitride or zirconium nitride. Surface topography was investigated with a high-resolution system for measuring surface finishes. Human gingival fibroblast (HGF) adhesion and proliferation were examined using MTT assay, Scanning Electron Microscopy (SEM) imaging, immunofluorescence analysis and real-time PCR for selected target genes. The hemolysis and AMES tests were performed to assess the chemical compounds’ blood compatibility and mutagenic potential, respectively. Antibacterial activity was tested against five bacterial strains isolated from the oral cavity (*Streptococcus salivarius*, *S*. *sanguinis*, *S*. *mutans*, *S*. *sobrinus*, *S*. *oralis*), and the percentage of dead bacteria was calculated. Roughness measurements confirmed a substantial similarity between the surfaces and their compatibility with clinical applications. MTT assay, SEM analysis and immunofluorescence staining showed adhesion and proliferation of HGFs cultured on all the examined surfaces. PCR confirmed that HGFs produced extracellular matrix components efficiently on all the surfaces. No hemolytic activity was detected, and the AMES test confirmed the surfaces’ clinical safety. For all tested bacterial strains, biofilms grown on the zirconium nitride surface showed a higher percentage of dead bacteria than on the other disks. The titanium nitride surface inactivated bacterial biofilms, too, but to a lesser extent.

## Introduction

Titanium (Ti) and Ti alloys abutments are commonly used in dental practice due to their biocompatibility and mechanical properties [1]. In particular, Ti6Al4V alloy is one of the most widely used material for dental implant abutments [2,3]. However, in presence of a thin gingival phenotype, grey Ti and Ti alloys abutments might become visible through soft tissue. To overcome these aesthetic issues and in order to achieve a better microbial seal, different surface modifications and materials have been developed [4–6].

The long-term success of dental implants largely depends on the surrounding soft tissues, which should provide a protective seal against bacterial colonization to prevent peri-implant disease [7,8].

An ideal transmucosal implant component should not only facilitate epithelial and connective tissue attachment and its maintenance over time, but also minimize bacterial colonization and plaque accumulation [9].

Studies have demonstrated that surface properties of dental implants have a great impact on early bacterial adhesion [10,11]. Early colonizers like Streptococci create an environment that also favors the accumulation of later colonizers, so for implant materials exposed to the oral cavity to inhibit early colonization is crucially important.

Plaque formation is largely influenced by parameters such as the surface roughness and chemical composition of abutments and the transmucosal portions of implants [12]. Rougher surfaces facilitate bacterial colonization and rapid biofilm maturation, whereas smoother surfaces provide a less suitable substrate for bacteria [11,13]. The transmucosal portion of a dental implant should not exceed a Ra value of 0.2 μm in order to contain plaque formation and favor a stable soft tissue seal around the abutment [13–15]. A Ti surface coating with a Ra ≤0.088 μm and Rz <1.027 μm reportedly strongly inhibited plaque accumulation and maturation *in vivo*, and this degree of smoothness was consequently suggested for transmucosal and healing implant components [16]. It has also been reported that surface roughness modulates cell behavior [17]. Investigating the relationship between surface roughness and fibroblast proliferation on Ti and Ti-based alloys, Ponsonnet et al. [18] found that cell proliferation was higher when the peak-to-valley roughness (Rz) was less than 1 μm.

Chemical composition influences the antibacterial activity and cell biocompatibility of the surfaces too. Coatings with nitrides such as zirconium nitride (ZrN) and titanium nitride (TiN) have attracted attention because they have been shown to limit bacterial colonization by comparison with other abutment materials used in clinical practice [19–21]. ZrN does not affect cell biocompatibility, as fibroblasts react biologically to ZrN coatings in the same way as to uncoated Ti [22]. ZrN has also been shown to facilitate human gingival fibroblast (HGF) attachment *in vivo* [9].

The aims of the present study were to characterize the topography of four different surface treatments on Ti disks (uncoated machined Ti6Al4V; anodized; TiN-coated; and ZrN-coated) and to compare them in terms of fibroblast proliferation activity and bacterial biofilm inactivation.

## Materials and methods

### Biomaterials

This *in vitro* study was conducted on machined titanium TiAl6V4, grade 5, disks (*Ti disks*) 13 mm in diameter, and 3 mm thick with different surface finishes, i.e. anodized (*ANODIZED*), coated with titanium nitride (*TiN*), or coated with zirconium nitride (*ZrN*), as compared with uncoated machined Ti disks (MACHINED), which were used as controls.

Anodization (MegaGen R&D center, MegaGen, South Korea) was accomplished by applying a current of 2.2 A at a voltage of 8V in a 5% phosphoric acid solution for 10 min. TiN (Medthin™, IonBond) and ZrN (Medthin™, IonBond) coatings were produced using physical vapor deposition (PVD) method.

### Surface topography analyses

Surface topography was investigated with a high-resolution system for measuring both profiles and areal features, in the form of a stylus profilometer (Form Talysurf i-Series, Taylor Hobson Ltd, Leicester, UK) with a 2 μm stylus tip radius.

Two disks were analyzed for each type of surface finish. An area– 2mm x 2mm–was scanned in both sides of each disk: a total of 201 profiles were acquired per single area. For 2D roughness profile analysis, 13 profiles were randomly extracted from the scanned area. For 3D roughness areal analysis, 4 independent sub-areas– 1mm x 1mm–were extracted.

The profile and areal data were filtered to eliminate waviness and form components of surface topography using a Gaussian filter with a sampling length of 0.25 mm, in accordance with ISO 4288 and ISO 25178.

The following parameters, from ISO 4287 and ISO 25178, were used to describe the surface topography:

- profile roughness parameter: Ra, Rz, Rsm, Rsk, and Rku
- areal parameters: Sa and Sz

Data were analyzed with Talymap software (Taylor Hobson Ltd, Leicester, UK).

### Cell cultures

HGFs were prepared according to a modified version of the Rheinwald and Green protocol [23]. The source of tissue was gingiva derived from patients undergoing oral surgeries, after they signed the informed consent in accordance with Italian Legislation and with the code of Ethical Principles for Medical Research involving Human Subjects of the World Medical Association (Declaration of Helsinki). Gingival fragments were obtained from the retromolar area as a consequence of the surgical flap regularization before suture after impacted third molar extraction or during gengivectomy. As they represent biological material waste, they are exempted from the local ethical board approval. Donor patients identifying data were not collected. After epithelial sheet dispase removal, gingiva was cut into small pieces (2–3 mm^2^) and fibroblasts were isolated by sequential trypsin and collagenase digestion. The HGFs were then cultured with complete DMEM (cDMEM), consisting of Dulbecco's Modified Eagle Medium (DMEM; EuroClone, Milan, Italy) supplemented with 10% Fetal Bovine Serum (FBS; Bidachem S.p.A., Milan, Italy), 2 mM L-glutamine (EuroClone), and 1% penicillin/streptomycin (EuroClone).

At confluence, the cells were harvested with a trypsin treatment. HGFs were detached from the culture plates and seeded onto the coated and uncoated Ti disks at a density of 2×10^4^ cells/disk in 12-well plastic tissue culture plates (Corning; VWR International, Milan, Italy). In detail, 50 μL of cDMEM containing the cell suspension were put directly onto each disk’s surface to allow cell adhesion. The disks were then incubated for 2 h at 37°C and 5% CO_2_ before adding 1 mL of culture medium. In order to maintain humidity, sterile Phosphate Buffered Saline (PBS; EuroClone) was added in the spaces between the wells of the culture plates. The cells were cultured in cDMEM at 37°C with 5% CO_2_ for up to 21 days, replacing the medium every 3 days.

### MTT assay

To establish the cell proliferation rate on the disks with the four different surfaces, the cytotoxicity assay based on methyl thiazolyl tetrazolium (MTT) was performed according to the method proposed by Denizot and Lang, with minor modifications [24]. The test is based on mitochondria viability, i.e., only functional mitochondria can oxidize an MTT solution, producing a typical blue-violet end product. After harvesting the culture medium, the cells were incubated for 3 h at 37°C in 1 mL of 0.5 mg/mL MTT solution prepared in PBS solution. After removing the MTT solution with a pipette, 0.5 mL of 10% dimethyl sulfoxide in isopropanol (iDMSO) was added for 30 min at 37°C. For each sample, optical density (O.D.) values at 570 nm were recorded in duplicate on 200 μL aliquots deposited in 96-well plates using a multilabel plate reader (Victor 3 Perkin Elmer, Milano, Italy). All samples were examined after 3, 7, 14 and 21 days of culture.

### Morphological analysis

For Scanning Electron Microscopy (SEM) imaging, HGFs grown on the different disk’s surfaces for 14 and 21 days were fixed in 2.5% glutaraldehyde prepared in 0.1 M cacodylate buffer for 1 h, then progressively dehydrated in ethanol. All micrographs were obtained using a JSM JEOL 6490 SEM microscope (JEOL, Tokyo, Japan). The SEM analysis was performed at Centro di Analisi e Servizi Per la Certificazione (CEASC, University of Padova, Padova, Italy).

### Hemolysis test

The procedure used to assess the blood compatibility of the coated and uncoated Ti disks (test materials) followed the standard practices set forth in ASTM F756. The extract method was chosen, using 0.9% saline as the extraction vehicle. Blood was obtained from healthy New Zealand rabbits for routine blood tests, with procedures carried out in the authorized Veterinary Hospital of Padova University. The animals were not euthanized and did not receive suffering, for this any approval-use of commitee or equivalent research ethics commitee was needed. Potassium oxalate was used as anticoagulant at a final concentration of 1.0 mg/mL. For the extraction of the test materials, a 2-g portion of test material was covered with 10 mL of saline. Duplicate preparations were extracted at 50°C for 72 h. For the negative control, a 30 cm^2^ portion of high-density polyethylene was covered with 10 mL of saline. For the positive control, a 10-mL portion of sterile water for injection was prepared. Both controls were exposed to the same extraction conditions as the test materials. Then 7 mL of the prepared extracts were added to 1 mL of rabbit blood, incubated for 60 min at 37°C, then centrifuged at 2500 rpm for 5 min. Next, a 1 mL aliquot of the supernatant obtained from the test materials, and from the negative and positive controls was added to 1 mL of Drabkin’s reagent (Sigma-Aldrich, St. Louis, MO, USA) and the O.D. was measured at 540 nm using the Victor 3 plate reader. The hemolysis rate (HR) was calculated from the mean O.D. for each group as follows:

HR(%)=(O.D.t–O.D.nc)/(O.D.pc–O.D.nc)×100%

where O.D.t is the absorbance of the test sample, and O.D.pc and O.D.nc that of the positive and negative controls, respectively. The results were interpreted as nonhemolytic if the HR was 2% or less, and hemolytic if HR exceeded 2%.

### Ames test

The Ames test was performed to assess the mutagenic potential of the coated and uncoated Ti disks using the Salmonella Mutagenicity Complete Test Kit (Moltox, Molecular Toxicology Inc., Boone, NC, USA). In detail, nutrient broth (blank) was used as the extraction vehicle, aluminum oxide ceramic rod (VITA In-Ceram Alumina CA-12 CE 0124 LOT 15320) as the negative control, and ICR 191 acridine (Moltox, 60–101) and sodium azide (Moltox, 60–103) as the positive controls. The extraction conditions were (24 ± 2) h at (37 ± 1)°C. Three replicates were run for the test samples and controls. The results were interpreted as follows: a surface was not mutagenic if the number of reverted colonies equated to those observed in the blank and negative controls; or it was mutagenic if the number of reverted colonies equated to those observed in the positive controls.

### RNA extraction and first-strand cDNA synthesis

Total RNA was extracted with the RNeasy Mini Kit (Qiagen, Hilden, Germany), including DNase digestion with the RNase-Free DNase Set (Qiagen), from cells seeded onto coated and uncoated Ti disks for 21 days. The RNA quality and concentration of the samples was measured with the NanoDrop^TM^ND-1000 (Thermo Fisher Scientific, Waltham, MA, USA).

For the first-strand cDNA synthesis, 800 ng of RNA was reverse-transcribed using an RT2 First Strand kit (Qiagen).

### Real-time PCR

Real-time PCR was performed according to the user’s manual for the **Cell Adhesion Molecules** RT2 Profiler PCR Array (SABiosciences, Frederick, MD, USA) and using RT2 SYBR Green ROX FAST Master Mix (Qiagen).

Genes present in the kit and analyzed were the following:

Transmembrane Receptors: CD44, CDH1 (E-Cadherin), HAS1, ICAM1, ITGA1, ITGA2, ITGA3, ITGA4(CD49D), ITGA5, ITGA6, ITGA7, ITGA8, ITGAL, ITGAM, ITGAV, ITGB1, ITGB2, ITGB3, ITGB4, ITGB5, MMP14, MMP15, MMP16, NCAM1, PECAM1, SELE, SELL (LECAM-1), SELP, SGCE, SPG7, VCAM1.

Cell-Cell Adhesion: CD44, CDH1 (E-Cadherin), COL11A1, COL14A1, COL6A2, CTNND1, ICAM1, ITGA8, VCAM1.

Cell-Extracellular Matrix (ECM) Adhesion: ADAMTS13, CD44, ITGA1, ITGA2, ITGA3, ITGA4 (CD49D), ITGA5, ITGA6, ITGA7, ITGA8, ITGAL, ITGAM, ITGAV, ITGB1, ITGB2, ITGB3, ITGB4, ITGB5, SGCE, SPP1, THBS3.

Other Cell Adhesion Molecules: CNTN1, COL12A1, COL15A1, COL16A1, COL5A1, COL6A1, COL7A1, COL8A1, VCAN, CTGF, CTNNA1, CTNNB1, CTNND2, FN1, KAL1, LAMA1, LAMA2, LAMA3, LAMB1, LAMB3, LAMC1, THBS1 (TSP-1), THBS2, CLEC3B, TNC, VTN.

Extracellular Matrix (ECM) Molecules

- Basement Membrane Constituents: COL4A2, COL7A1, LAMA1, LAMA2, LAMA3, LAMB1, LAMB3, LAMC1, SPARC.
- Collagens & ECM Structural Constituents: COL11A1, COL12A1, COL14A1, COL15A1, COL16A1, COL1A1, COL4A2, COL5A1, COL6A1, COL6A2, COL7A1, COL8A1, FN1, KAL1.
- Extracellular Matrix (ECM) Proteases: ADAMTS1, ADAMTS13, ADAMTS8, MMP1, MMP10, MMP11, MMP12, MMP13, MMP14, MMP15, MMP16, MMP2, MMP3, MMP7, MMP8, MMP9, SPG7, TIMP1.
- Extracellular Matrix (ECM) Protease Inhibitors: COL7A1, KAL1, THBS1 (TSP-1), TIMP1, TIMP2, TIMP3.
- Other Extracellular Matrix (ECM) Molecules: VCAN, CTGF, ECM1, HAS1, SPP1, TGFBI, THBS2, THBS3, CLEC3B, TNC, VTN.

Thermal cycling and fluorescence detection were performed using a Rotor-Gene Q 100 (Qiagen).

The data were analyzed using Excel-based PCR Array Data Analysis Templates (SABiosciences).

The thermal cycling conditions were as follows: 15 min denaturation at 95°C; then 40 cycles of denaturation for 15 s at 95°C; annealing for 30 s at 60°C; and elongation for 20 s at 72°C. Values were normalized (2ΔCt) to the expression of the glyceraldehyde-3-phosphate dehydrogenase (GAPDH) internal reference, the abundance of which remained constant under our experimental conditions. The experiments were repeated 3 times.

### Immunofluorescence (IF) analysis

For IF staining, cells grown on the different disks for 14 days were fixed in 4% paraformaldehyde solution in PBS for 10 min, then permeabilized for 10 min in 0.2% Triton X-100 (Sigma-Aldrich) prepared in PBS. After three washing with PBS, the cells were incubated in 2% Bovine Serum Albumin (BSA; Sigma-Aldrich) solution in PBS for 1 h at room temperature. The cells were then incubated overnight at 4°C with the Monoclonal Anti-Vinculin primary antibody produced in mouse (Sigma-Aldrich), diluted 1:400 in 1% BSA solution. IF staining was performed with the DyLight™ 488-Labeled anti-mouse IgG secondary antibody (KPL, Gaithersburg, MD, USA) diluted 1:100 in 1% BSA for 1 h at room temperature. Alternatively, the cells were stained with 5 μg/mL Phalloidin (Sigma-Aldrich) for 40 min at room temperature. Nuclear staining was performed with 2 μg/mL Hoechst H33342 (Sigma-Aldrich) solution for 15 min. The cells were coverslipped with a drop of mounting medium, then observed with the LeicaDM5000 B microscope (Imaging Facility, Department of Biology, University of Padova, Padova, Italy).

### Bacterial strains and biofilm quantification

*Streptococcus salivarius*, *S*. *sanguinis*, *S*. *mutans*, *S*. *sobrinus*, and *S*. *oralis* isolated from clinical specimens and identified by mass spectrometry were routinely maintained on brain-heart infusion (BHI; BD Difco, Milan, Italy) agar plates. To assess biofilm formation, bacteria were grown in BHI broth overnight at 37°C, then plated (1x10^7^ CFU/mL) on the previously-sterilized, coated and uncoated Ti disks placed in 24-well plates. Bacteria were grown in a total volume of 2 mL of BHI broth for 120 h at 37°C without agitation.

After incubation, the Ti disks were washed in 0.85% NaCl to remove loosely bound bacterial cells. Biofilms growing on the surface of the Ti disks were gently removed with cell scrapers (Corning), transferred into separate tubes, and stained with the LIVE/DEAD^®^ BacLight^TM^ Bacterial Viability Kit (Thermo Fisher Scientific) for 15 min at room temperature in the dark. Briefly, the samples were incubated with 5 μM SYTO9 (a green fluorescent nucleic acid dye labeling both live and dead bacteria), and 30 μM propidium iodide, penetrating only bacteria with damaged membranes. Unlabeled dyes were removed by washing and the bacteria were analyzed using flow cytometry in a BD FACSCalibur (Becton Dickinson, Franklin Lakes, NJ, USA), setting the signal discriminator at 15% of the log-integrated green fluorescence, and acquiring 5x10^4^ bacterial cells at a rate 300 particles/second. Data were analyzed with CellQuest software (Becton Dickinson) and the percentage of dead bacteria (i.e. bacteria with a high propidium iodide fluorescence and a low SYTO9 green fluorescence) was recorded.

### Statistical analysis

One-way analysis of variance (ANOVA) was used for the data analysis. A repeated-measures ANOVA was run with a post-hoc analysis using Bonferroni’s correction for multiple comparisons, and t-tests were used to ascertain significant differences (P<0.05). Repeatability was calculated as the standard deviation of the difference between measurements. All testing was done using SPSS 16.0 software (SPSS Inc.; Chicago, IL, USA) (licensed by the University of Padova).

## Results

### Surface topography analyses

The aim of the surface topography investigations was to analyze the surface roughness of the disks in order to compare it with data in the literature and to discriminate the surface roughness of the anodized, TiN and ZrN samples from their original (i.e. machined) morphology. The results of the surface texture analysis are shown in Tables 1 and 2. The samples had mean Ra values within the range from 0.067 (SD 0.008) μm, relevant to ZrN, to 0.115 (SD 0.022) μm, relevant to TiN. Ra represented the principal parameter under investigation, differentiating coated (anodized, TiN and ZrN) from machined samples. Furthermore, there were significant differences in Ra values between ZrN samples and the other coated samples, anodized and TiN-coated. Between the latters no significant difference in Ra values was found. Rsm, the mean width (i.e. wavelength) of the profile, was found to be significant similar among all samples: it is worth noting that Rsm values are strictly related to the machining (i.e. turning) feed rate while it is minimally sensitive to subsequent surface treatments, thus confirming that the manufacturing process was the same for all the samples.

**Table 1:** Topographical characterization (profile roughness parameters).

Samples	Ra (SD) μm (Lc = 0.25mm)	Rz (SD) μm (Lc = 0.25mm)	Rsm (SD) mm (Lc = 0.25mm)	Rsk (SD) (Lc = 0.25mm)	Rku (SD) (Lc = 0.25mm)
MACHINED	0.089 (0.008)	0.664 (0.051)	0.116 (0.012)	-0.095 (0.207)	3.485 (0.411)
ANODIZED	0.113 (0.004)	0.739 (0.098)	0.127 (0.018)	-0.280 (0.389)	3.176 (0.709)
TiN	0.115 (0.022)	0.957 (0.156)	0.118 (0.012)	0.083 (0.243)	4.437 (1.254)
ZrN	0.067 (0.008)	0.754 (0.091)	0.114 (0.012)	0.603 (0.627)	8.245 (3.200)

**Table 2:** Topographical characterization (areal parameters).

Samples	Sa (SD) μm (Lc = 0.25mm)	Sz (SD) μm (Lc = 0.25mm)
MACHINED	0.080 (0.010)	1.404 (0.333)
ANODIZED	0.065 (0.010)	1.031 (0.382)
TiN	0.079 (0.029)	1.846 (0.700)
ZrN	0.073 (0.011)	1.713 (0.258)

The Rz parameter, describing the maximum peak-to-valley depth, was used in the Rz/Ra ratio for its sensitivity to surface modifications: relevant differences were assessed, which were confirmed by Rsk and Rku, skewness and kurtosis of 2D profiles.

As compared to the other coated surfaces, ZrN coating showed major changes in profile from the original surface topography, with Rz/Ra 7.5, Rsk -0.095 (SD 0.207) and Rku 3.485 (SD 0.411) before coating (MACHINED) and Rz/Ra 11.2, Rsk 0.603 (SD 0.627) and Rku 8.245 (SD 3.200) after coating.

**Fig 1:**
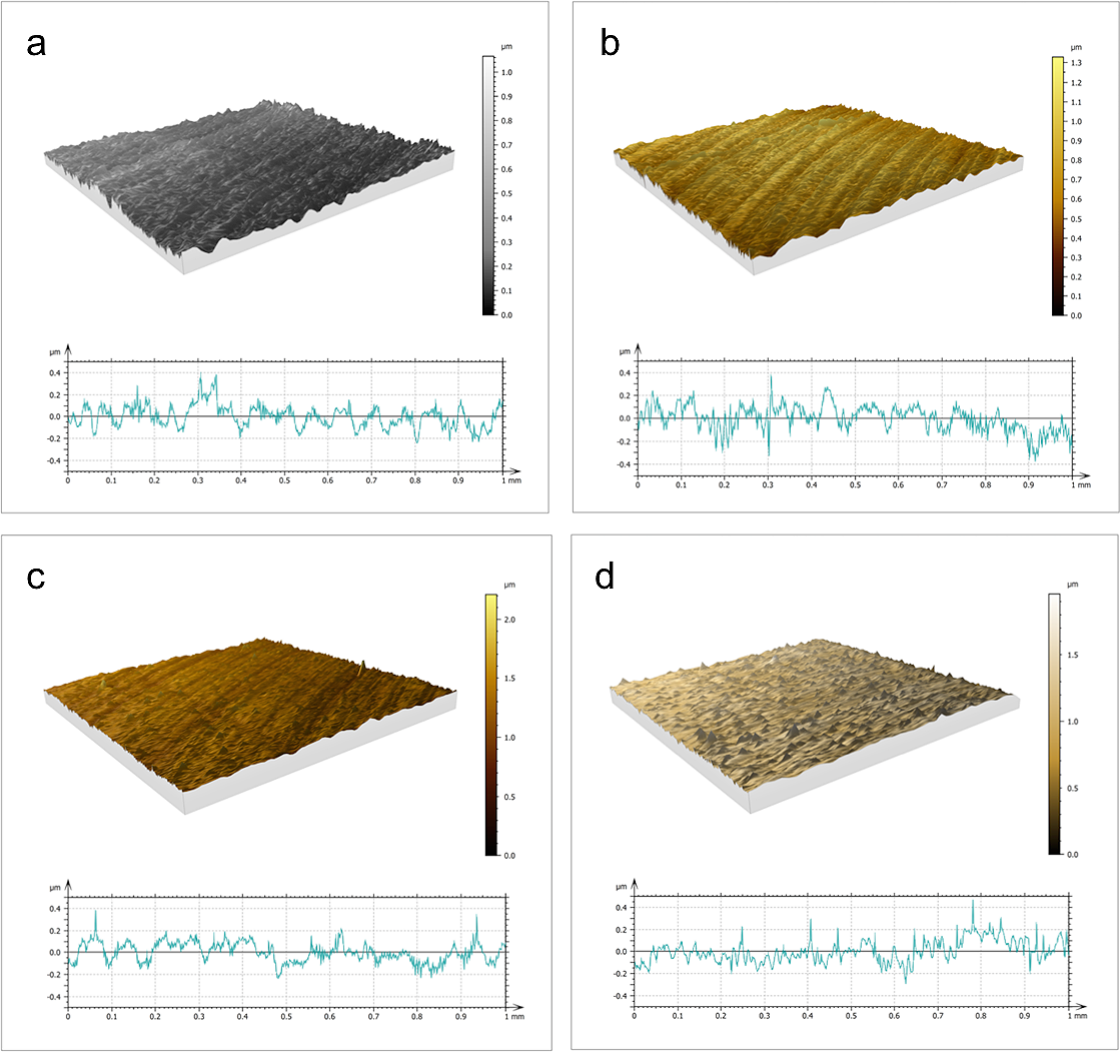
3D maps and related 2D profiles. 3D maps and related 2D profiles of: a) machined, b) anodized, c) TiN-coated, and d) ZrN-coated disks.

3D mapping was performed in order to confirm significant differences in 2D analysis, though this is not generally reported in the specific literature (Fig 1). 3D mean roughness (Sa) values were lower than the 2D parameters in the related samples. Differences seemed significant in Sz, where such an extended range of values, from 1.031 to 1.846 μm, would suggest the need for further investigations.

### Proliferation

**Fig 2:**
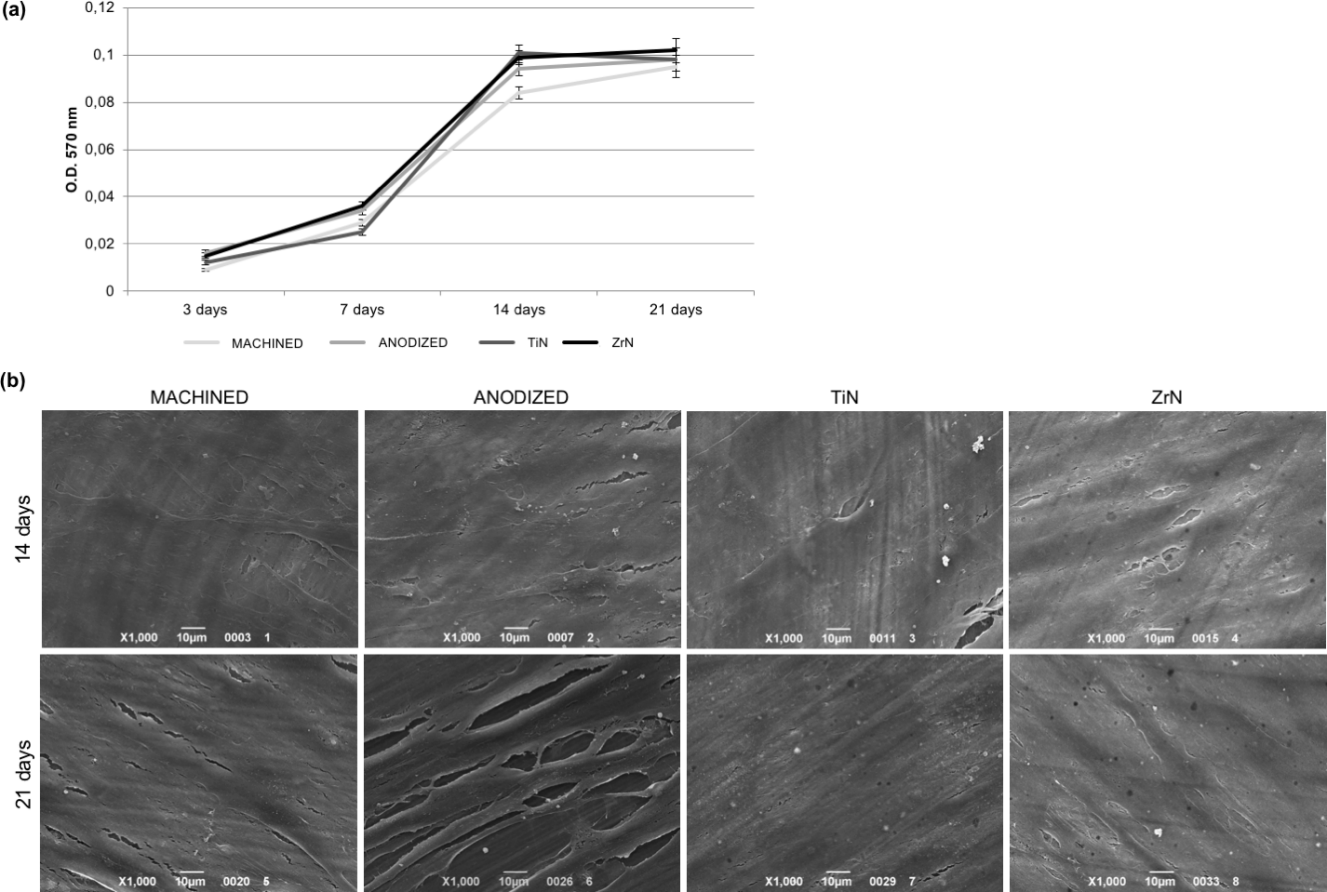
Proliferation of fibroblasts seeded on Ti disks. (**a**) MTT assay of HGFs cultured on the different Ti disks for 3, 7, 14 and 21 days shows that cells are vital and able to proliferate on all the surfaces examined. (**b**) SEM images (1000x magnification) of HGFs grown onto the four Ti disks reveal that cells reached confluence after 14 days from seeding, forming a continuous monolayer, which persisted up to 21 days.

The biocompatibility of the Ti disks was tested in terms of the proliferation rate of fibroblasts seeded onto the disks at different times by means of the MTT test. The HGFs were able to adhere to the disks and proliferate. As shown in Fig 2A, the cell proliferation increased over the course of time up to 14 days in culture. After 21 days from seeding, cell proliferation was found to be nearly the same level as that seen at 14 days, thus indicating that cell culture reached confluence. No significant differences emerged between the types of disk surface. The cell colonization of the samples is evident from the SEM images. As shown in Fig 2B, after 14 days from seeding, HGFs were found to be well spread and attached on the surface of the samples, forming a continuous monolayer. Then, the cells reached confluence and stopped proliferating, as shown in images taken at 21 days of culture.

### Hemolysis test

This test was used to assess the blood compatibility of the Ti disks, since they are intended for use in contact with the blood. The test measures the free hemoglobin released into the plasma when blood cells are damaged. While the samples were incubated with diluted blood, the blood cells could release hemoglobin into the plasma. The product of this reaction was quantified by measuring the OD at 540 nm with a spectrophotometer. As shown in Table 3, no hemoglobin was detected in any of the samples, meaning that the tested materials have no hemolytic activity.

**Table 3:** Hemolysis test.

Sample	O.D. (SD)	HR (%)	Interpretation
Positive control	0.8752 (0.022)	100	Hemolytic
Negative control	0.0132 (0.004)	0	Nonhemolytic
MACHINED	0.0142 (0.002)	0.116	Nonhemolytic
ANODIZED	0.0135 (0.002)	0.035	Nonhemolytic
TiN	0.0142 (0.002)	0.116	Nonhemolytic
ZrN	0.0136 (0.002)	0.046	Nonhemolytic

### Ames test

The clinical safety of the implant materials was also assessed in terms of their mutagenic potential by means of the Ames test. A positive test result indicates that the chemical is mutagenic and may therefore act as a carcinogen (since cancer is often associated with mutations). The Ames test uses several strains of the bacterium *Salmonella typhimurium* carrying mutations in genes involved in histidine synthesis. The tester strains are especially designed to detect either frame shift (e.g. strains TA-1537 and TA-1538) or point (e.g. strain TA-1531) mutations in the genes required to synthesize histidine, so mutagens acting via different mechanisms may be identified. The tester strains also carry mutations in the genes responsible for lipopolysaccharide synthesis (making the cell wall of the bacteria more permeable), and in the excision repair system to make the test more sensitive. The mutagenicity of a substance is proportional to the number of colonies observed. As shown in Table 4, no mutagenic activity was revealed for any of the surfaces tested.

**Table 4:** Ames test.

	Tester strain							
	STDisc™: TA1535		STDisc™: TA1537		STDisc™: TA98		STDisc™: TA100	
Sample	Number of colonies / plate *	Result	Number of colonies / plate *	Result	Number of colonies / plate *	Result	Number of colonies / plate *	Result
Blank	4 (3)	NM	5 (3)	NM	5 (3)	NM	3 (3)	NM
Negative control	3 (2)	NM	4 (2)	NM	2 (2)	NM	4 (2)	NM
Positive control ICR191 (60–101)	932 (86)	M	931 (77)	M	926 (73)	M	929 (78)	M
Positive control Sodium Azide (60–103)	849 (52)	M	858 (53)	M	846 (54)	M	839 (51)	M
MACHINED	2 (2)	NM	3 (2)	NM	2 (2)	NM	3 (2)	NM
ANODIZED	2 (1)	NM	3 (2)	NM	4 (2)	NM	2 (2)	NM
TiN	2 (2)	NM	3 (2)	NM	4 (2)	NM	3 (2)	NM
ZrN	3 (2)	NM	4 (2)	NM	3 (2)	NM	2 (2)	NM

NM: not mutagenic; M: mutagenic.

*: (mean (SD), n = 3).

### Gene expression of cell adhesion and proliferation markers

**Fig 3:**
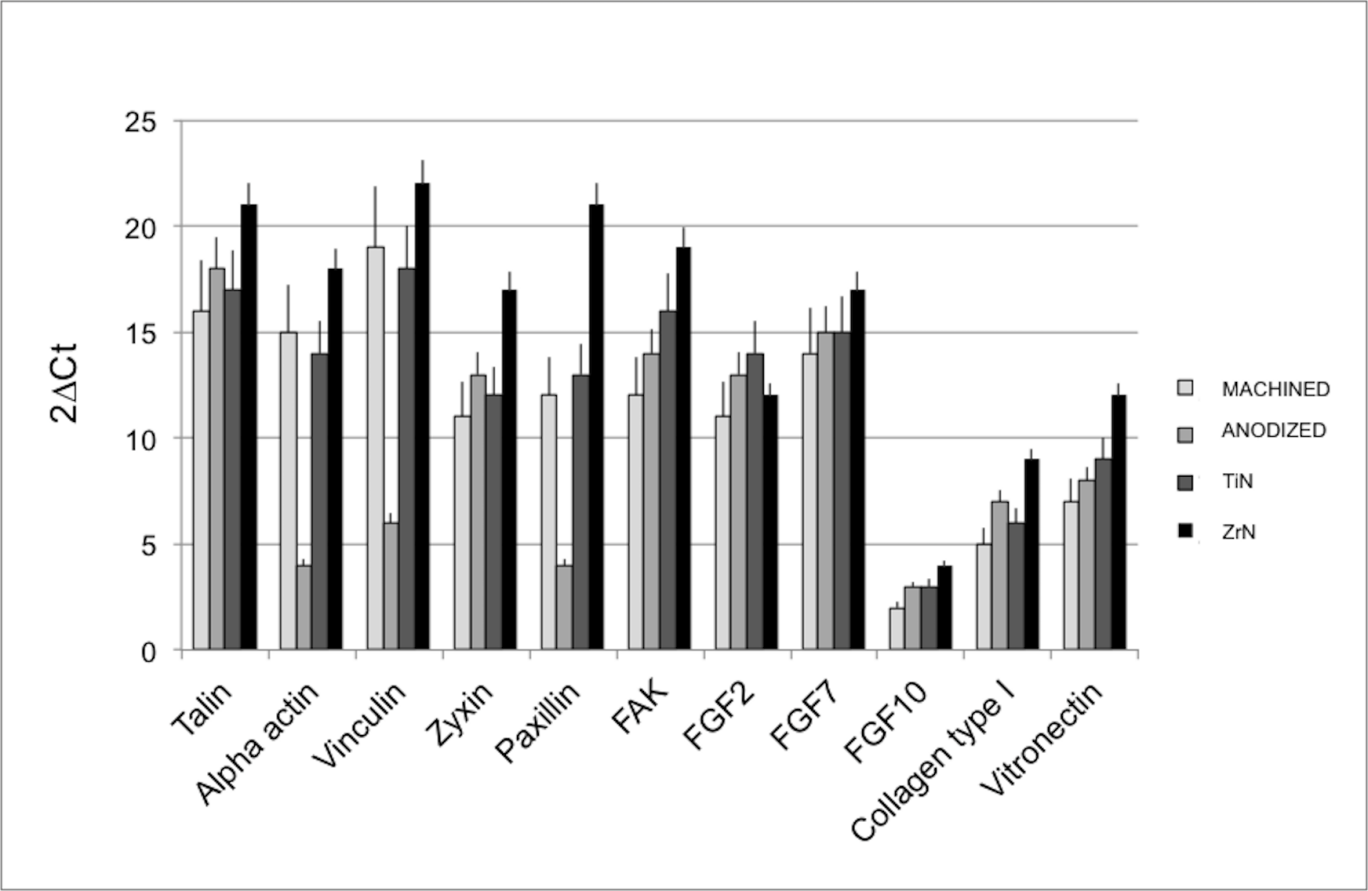
Real-time PCR analysis. Real-time PCR analysis of cell adhesion and proliferation markers in HGFs cultured on Ti disks for 21 days. Results for each experiment are obtained from triplicate experiments and values are expressed as the mean ± SD.

Gene expression techniques were used to test the influence of the different surfaces on fibroblast adhesion and proliferation (Fig 3). The genes considered were talin, alpha-actinin, vinculin, zyxin, paxillin, vitronectin, focal adhesion kinase (FAK), and collagen type I, all involved in cell adhesion. Good mRNA relative expression levels were found on all the surfaces examined, but the highest gene expression values were observed on the ZrN-treated disks. Focusing on the expression of fibroblast growth factors (FGF) 2, 7 and 10, the fibroblasts were able to produce these growth factors when seeded on all the coated and uncoated Ti surfaces, again with the highest levels on the ZrN disks.

### IF analysis

**Fig 4:**
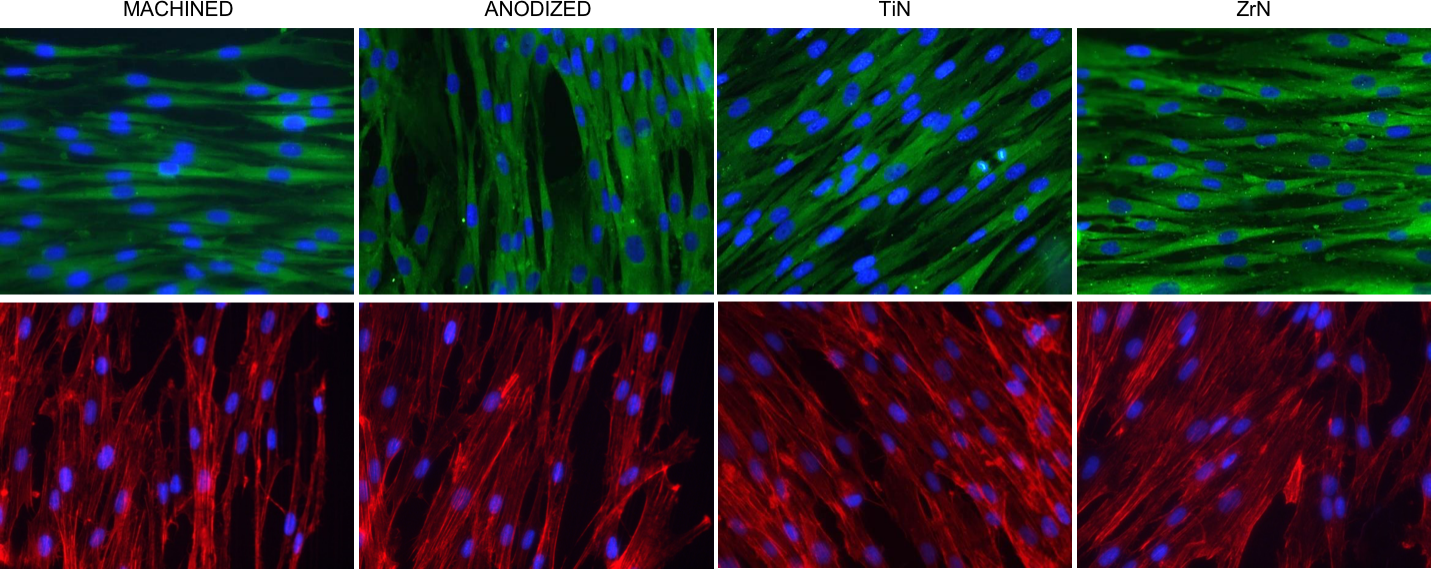
IF analysis. IF staining of HGFs grown on Ti disks for 14 days. Upper panels display staining of vinculin (in green); lower panels show labeling of actin filaments by Phalloidin (in red). For both panels, nuclei are stained blue. Images taken at 40x magnification.

In order to evaluate the adhesion of HGFs on the different Ti surfaces, IF staining of vinculin, one of the key players driving the formation of focal adhesion complexes [25], was performed. Upper panels in Fig 4 show positive staining for vinculin, confirming the ability of HGFs to adhere on all the surfaces examined. Since vinculin represents the major link between focal adhesions and the actin filaments, labeling of actin cytoskeleton was additionally performed [25]. As shown in lower panels of Fig 4, also in this case the cells were found to be adhered on the Ti disks without any significant difference between the surfaces.

### Bacterial strains and biofilm quantification

**Fig 5:**
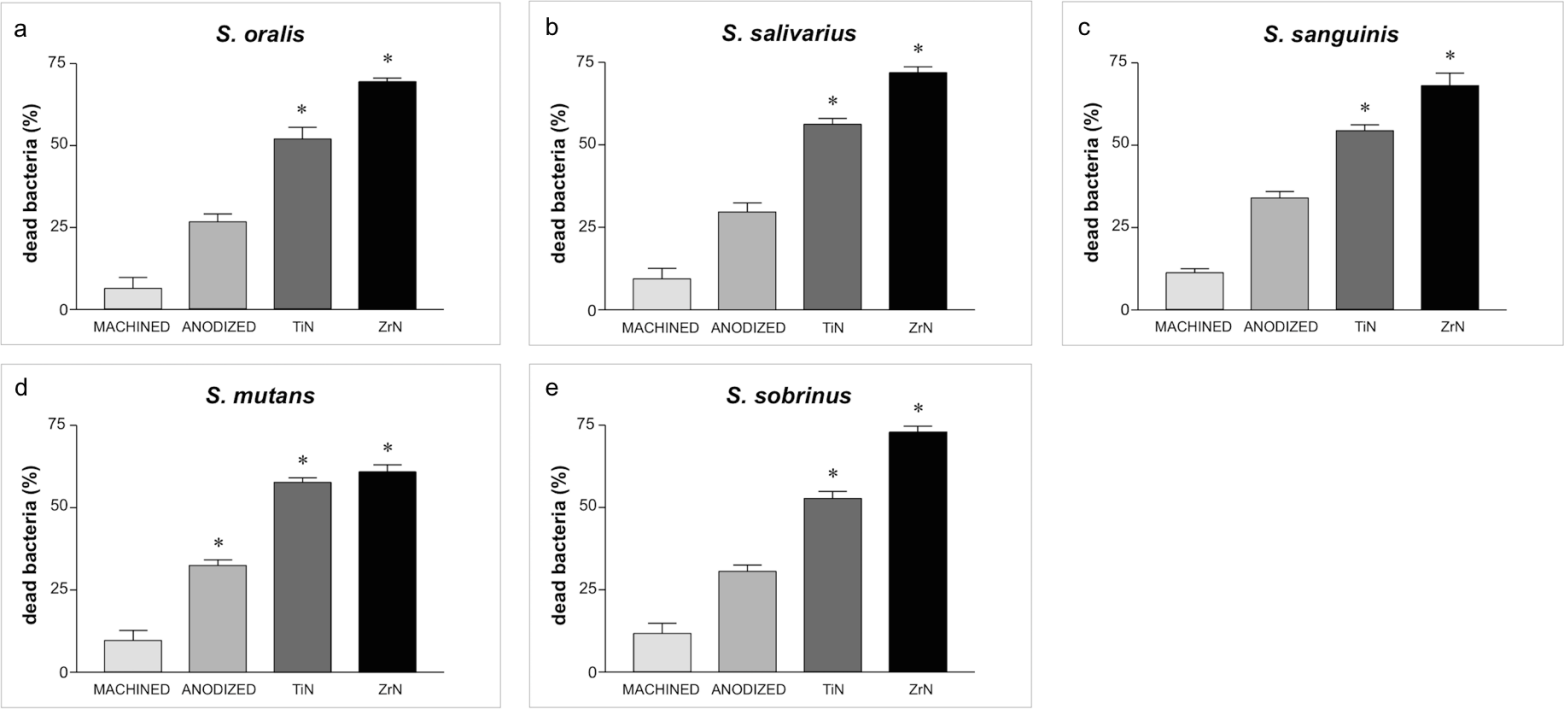
Percentage of dead bacteria. Percentage of dead bacteria in five strains grown on disks after 120 hours of incubation: a) *S*. *oralis*; b) *S*. *salivarius*; c) *S*. *sanguinis*; d) *S*. *mutans*; e) *S*. *sobrinus*.

The percentage of dead bacteria was higher in the biofilms grown on the TiN- and ZrN-coated disks than on the uncoated disks (Fig 5). For instance, the percentage of dead *S*. *oralis* on the ZrN- and TiN- treated disks was 69.45 ± 1.15 and 51.84 ± 3.73, respectively, whereas the uncoated surfaces only reduced the vitality of these bacteria by 6.4 ± 3.38 per cent (P<0.001). Similar results emerged for the other bacterial strains, so the antibacterial activity of the surface coatings was not limited to one bacterial species, it extended to several pathogens involved in peri-implant infections. As for the uncoated surfaces, the anodized Ti disks reduced bacterial bioavailability slightly better than the control disks, with a difference that nearly reached statistical significance (e.g. *S*. *mutans*).

## Discussion

Zirconium nitride (ZrN) was introduced as a coating material to improve the biocompatibility, antibacterial activity, and aesthetics of dental implant abutments [19–20, 26–27]. The present work confirmed that none of the surfaces investigated here have any mutagenic or hemolytic activity, so they are all safe to use in dental practice.

The MTT assay identified no significant differences between the surfaces considered–a finding consistent with other reports. For instance, MTT activity and total protein were significantly higher in fibroblasts cultured on a TiN-coated surface (Ra 0.19 μm) than on an uncoated polished Ti surface of similar roughness [28]. Chang et al. [22] also found no significant differences in this respect between uncoated and ZrN-coated Ti samples. These data are also consistent with another study [9], in which glass sheets coated with Ti, TiN or ZrN (average roughness 0.03–0.1 μm) appeared to facilitate HGF adhesion *in vivo*.

Fibroblast adhesion and spreading on all the investigated surfaces at different time points were demonstrated by SEM images. A more detailed analysis of the molecular mediators involved in cell adhesion and proliferation was performed using real-time PCR. Cell adhesion to the extracellular matrix (ECM) is mediated by the integrin family of heterodimeric receptors. ECM is a complex and dynamic network of macromolecules with different physical and biochemical properties, supporting cell organization and growth. The fundamental role of ECM in several key aspects of cell biology becomes increasingly evident since all cell types are in contact with it. ECM supports organ development, function and repairing. Physical properties such as topography, porosity, rigidity, and insolubility can influence different anchorage-related biological functions, like stem cell commitment, cell proliferation, and cell migration. Moreover, since it can act directly by binding cell surface receptors or by non-canonical growth factor presentation, ECM displays both direct and indirect signaling properties influencing cell behavior. On bone remodelling and regeneration process, mechanotransduction related to the ECM physical properties is essential, since it helps cells to sense the external forces and respond to the environment in an appropriate manner. It is therefore not surprising that alterations in a specific extracellular component can have a remarkable impact on the biochemical, biomechanical and physical properties of the ECM, leading to disorganized network and ultimately failure of organ homeostasis and function. Integrin receptors engaging with extracellular molecules give rise to the formation of multi-protein complexes that link the ECM to the cytoplasmic actin cytoskeleton. These adhesive structures are dynamic, often heterogeneous, and vary in size and organization. Signaling through these complexes and focal adhesions has been implicated in the regulation of several cellular processes, such as growth-factor-induced mitogenic signals, cell survival, proliferation and migration [29]. We confirmed the development of focal adhesion on all the surfaces tested, as revealed by the expression of the adapter proteins talin and paxillin, and their maturation into larger, stable complexes through the expression of zyxin and FAK. Once fibroblasts are well-anchored, they can proliferate to colonize the space, as demonstrated by the high FGF levels produced on all the surfaces considered, and the ZrN-coated disks in particular. Type I collagen is a major ECM component of fibroblasts. Real-time PCR data did not identify mRNA levels of type I collagen on uncoated Ti comparable with those found on ZrN-coated samples, as seen in Chang et al. [22]. In the present study, the highest levels of collagen type I, FGFs, and integrin-related proteins were all exhibited when cells were cultured on Ti disks coated with ZrN.

Consistent with real-time PCR findings, IF staining of vinculin, which plays a key role in stabilizing focal adhesion, confirmed HGF adhesion to all the coated and uncoated surfaces.

The development of surface coatings capable of minimizing the number of bacteria initially adhering to transmucosal implant components has become increasingly important [20, 30]. *Actinomyces* species and *Streptococci* are early colonizers that create a favorable environment for the arrival of late colonizers known to be involved in the pathogenesis of peri-implant disease [20, 31–32]. In an *in vivo* study, *Streptococci* were the predominant organisms in all the implanted surfaces examined [26].

In the present study the antibacterial activity of the surfaces was tested against five different strains of *Streptococci* isolated from the oral cavity (*Streptococcus salivarius*, *S*. *sanguinis*, *S*. *mutans*, *S*. *sobrinus*, *S*. *oralis*). ZrN and, to a lower extent, TiN appeared to be the most effective against all five bacterial strains. These findings are consistent with the results of previous *in vitro* and *in vivo* studies, in which bacterial colonization was more limited on ZrN-coated surfaces than on other abutment materials [20–21, 26]. In an *in vitro* study [19], for instance, the number of bacterial colonies (*Streptococcus sanguinis*, *Streptococcus mutans*) adhering to titanium disks was significantly lower for those with a TiN or ZrN coating than for a polished titanium surface, whether the disks were wetted with saliva or not. On examining the biofilm composition on three different surfaces after 60 h of intraoral exposure, Gröessner-Schreiber et al. [20] likewise found higher bacterial cell counts on glass surfaces coated with pure titanium than on glass specimens coated with TiN or ZrN.

ZrN-coated glass and titanium samples also showed the lowest values in terms of species richness, when compared with pure glass and polished titanium controls, after 24 h of intraoral exposure. No *Streptococcus* species were detected on ZrN-coated glass, and only two *Streptococcus* species were identified on ZrN-coated titanium surfaces [26].

In another study, ZrN-coated glass, ZrN-coated polished titanium, and uncoated polished Ti disks were mounted on removable intraoral splints in one adult. Short (24-hour) and long (14-day) incubations resulted in a significantly different composition of the bacterial biofilms on the ZrN-coated surfaces, suggesting that the chemical properties of the implanted surfaces attracted fewer pathogenic bacteria, thus reducing the risk of peri-implantitis [33].

The level detected in the peri-implant sulcus fluid of the active form of matrix metalloproteinase-8 (MMP-8), which rises in the case of peri-implantitis, was also found to differ significantly between titanium and ZrN abutment surfaces *in vivo* [34]. The authors concluded that ZrN-coated abutments have a beneficial effect on the collagenolytic tissue destruction driven by MMP-8 *in situ*.

Our results are at odds with the report from Chang et al. [22], who found that a ZrN coating offered no advantage over uncoated Ti, since the ZrN coating failed to prevent the growth of *S*. *aureus* on the surface of the plates *in vitro*. Kertzman et al. [35] also found no significant difference in bacterial load between uncoated and ZrN-coated Ti substrates, after incubating the samples with three strains of bacteria (*S*. *epidermis*, *S*. *aureus*. and *E*. *coli*).

Microbial adhesion to biomaterials has been related to surface roughness, which has been shown to influence the microbiology of supra- and subgingival plaque, justifying the use of smooth surfaces for abutment materials to reduce bacterial colonization and proliferation [14, 36]. Bacterial adhesion no longer seems to be affected below an average roughness (Ra) <0.2 μm because most bacteria are >0.2 μm in size [14–15]. When the effect of the surface roughness and hydrophobicity of the titanium samples on the adhesion of *P*. *gingivalis* was assessed *in vitro* [37], the results largely concurred with those of Bollen et al. just mentioned above [15], but the level of surface roughness capable of reducing bacterial adhesion was lower (between 0.034 and 0.155 μm) [37]. In the present study, all the samples investigated had Ra values between 0.067 and 0.115 μm. Our roughness measurements confirmed that the four surfaces considered were substantially similar in this respect, so it was probably not the main factor influencing bacterial adhesion. The antibacterial activity of the different surfaces depended largely on the chemical modifications of the titanium, with ZrN and TiN coatings exhibiting better results. Franková et al. [38] also demonstrated that ZrN-coated titanium samples, with Ra value of 0.28 μm, reduced the adhesion of *Streptococcus* strains significantly more than other chemically and physically modified titanium disks. The same authors judged that a combination of smoothness and chemical modification was responsible for the antibacterial activity of the ZrN-coated samples.

As peri-implantitis is associated with many bacterial species [39], further studies should be conducted testing a broader range of bacterial species.

Taken together, these results support a positive effect of ZrN coatings on fibroblast adhesion and proliferation, and an antibacterial activity against the main early colonizer bacteria *in vitro*.

## Conclusions

In conclusion, all the surfaces examined in this study proved safe for use as implant abutments. Surface roughness was highly smooth in all the samples, and the low variability in roughness among the different surfaces allowed to evaluate the effect of each material on fibroblast and bacteria adhesion and proliferation. When the surfaces were compared, however, the titanium disks with a zirconium nitride coating exhibited not only a greater cell biocompatibility, but also a greater antibacterial activity against five different bacterial strains.
